# Effects and Mechanisms of Chinese Herbal Medicine in Ameliorating Myocardial Ischemia-Reperfusion Injury

**DOI:** 10.1155/2013/925625

**Published:** 2013-10-31

**Authors:** Qing Liu, Jiqiang Li, Jing Wang, Jianping Li, Joseph S. Janicki, Daping Fan

**Affiliations:** ^1^Department of Cell Biology and Anatomy, University of South Carolina School of Medicine, Columbia, SC 29208, USA; ^2^The Second Clinical School of Medicine, Guangzhou University of Chinese Medicine, Guangzhou 510405, China

## Abstract

Myocardial ischemia-reperfusion (MIR) injury is a major contributor to the morbidity and mortality associated with coronary artery disease, which accounts for approximately 450,000 deaths a year in the United States alone. Chinese herbal medicine, especially combined herbal formulations, has been widely used in traditional Chinese medicine for the treatment of myocardial infarction for hundreds of years. While the efficacy of Chinese herbal medicine is well documented, the underlying molecular mechanisms remain elusive. In this review, we highlight recent studies which are focused on elucidating the cellular and molecular mechanisms using extracted compounds, single herbs, or herbal formulations in experimental settings. These studies represent recent efforts to bridge the gap between the enigma of ancient Chinese herbal medicine and the concepts of modern cell and molecular biology in the treatment of myocardial infarction.

## 1. Introduction

Myocardial infarction (MI) and the accompanying acute loss of viable myocardium is the leading cause of death in industrialized countries. Even if the patient survives the acute phase of MI, the subsequent adverse myocardial remodeling and impairment of cardiac function severely impact their quality of life and 5-year survival. Early restoration of blood flow to the ischemic myocardium is a common treatment strategy aimed at limiting myocardial infarct size. However, reperfusion can cause additional cell death and, in many cases, paradoxically increase infarct size, a situation referred to as myocardial ischemia-reperfusion (MIR) injury. MIR is characterized by a rapid increase in cytokines and chemokines and an influx of leukocytes into the vulnerable region bordering the infarcted site. This inflammatory response not only results in cardiomyocyte apoptosis during the acute phase, but also results in an adverse myocardial remodeling that further compromises cardiac function. Therefore, limiting ischemia-reperfusion (I/R) induced myocardial inflammation may not only lower the acute death rate, but also improve long term survival and quality of life [1]. Chinese herbal medicine, especially combined herbal formulations, has been widely used in traditional Chinese medicine for the treatment of MI for hundreds of years. The purpose of this review is to highlight recent studies that experimentally address the mechanistic effects of extracted compounds, single herbs, or herbal formulations on several factors and pathways known to be involved in MIR injury.

## 2. Myocardial Ischemia-Reperfusion Injury

### 2.1. Oxidative Stress

**Figure 1:**
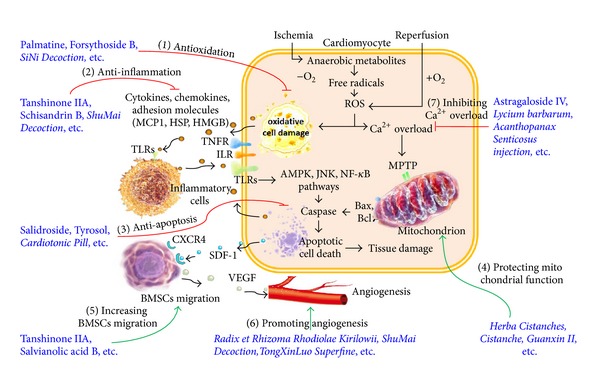
Effects and mechanisms of Chinese herbal medicine in myocardial ischemia-reperfusion (MIR) injury. During ischemia, oxygen is not available to accept the electrons in the metabolic degradation of substrates, and consequently anaerobic metabolites become important in the preservation of myocardial viability. However, free radicals and reactive oxygen species (ROS) formation is markedly increased in this procedure. Reperfusion also generates high ROS levels which have an adverse impact on specific signal transduction systems, thereby predisposing the heart to further oxidative cell damage. Damaged cell debris, fibrinogen, cytokines, and chemokines will activate the receptors, including TLRs, TNFR, and ILR, in the host inflammatory cells as well as the cardiomyocytes. This sterile inflammatory process leads to the formation of a vicious circle, whereby the cardiomyocyte TLRs, TNFR, and ILR are activated by inflammatory cell-generated ligands. Typically, this has an adverse impact on specific signal transduction systems (e.g., AMPK, JNK, and NF-*κ*B pathways), thereby activating the caspase cascade. Elevated ROS levels also result in intracellular Ca^2+^ overload which adversely affects mitochondrial function by opening the mitochondrial permeability transition pore (MPTP). As a result, the balance between Bax and Bcl is interrupted and the caspase cascade is further activated, leading to apoptotic cell death and myocardial tissue damage. Injured tissue expresses SDF-1, which interacts with its specific receptors (e.g., CXCR4) to facilitate the trafficking, adhesion, and infiltration of bone marrow derived stem cells (BMSCs). BMSCs produce high levels of the endothelial cell-specific angiogenic factor, VEGF, which is a critical regulator of angiogenesis that includes the stimulation of proliferation, migration, and proteolytic activity of endothelial cells and eventually leads to an increase in vessel density and the facilitating of myocardial regeneration and remodeling. During the MIR injury process, there are seven target areas where Chinese herbal medicine can exert protective effects on cardiomyocyte. Examples are as follows: (1) anti-oxidation actions of Palmatine, Forsythoside B, and SiNi Decoction; (2) anti-inflammatory properties of Tanshinone IIA, Schisandrin B, and ShuMai Decoction; (3) anti-apoptosis ability of Salidroside, Tyrosol, and Cardiotonic Pill; (4) protection of mitochondrial function by Herba Cistanches, Cistanche, and Guanxin II; (5) increasing BMSCs migration by Tanshinone IIA and Salvianolic acid B (6) promoting angiogenesis by Radix et Rhizoma Rhodiolae Kirilowii, ShuMai Decoction and TongXinLuo Superfine; and (7) inhibiting Ca^2+^ overload by Astragaloside IV, *Lycium barbarum*, and *Acanthopanax* senticosus injection.

Reactive oxygen species (ROS) have both a physiological and pathological role in cellular and tissue adaptation to environmental factors. Normally, low levels of oxygen radicals and oxidants are present in cells and are important in maintaining cellular homeostasis, mitosis, differentiation, and signaling [2]. However, during MIR, ROS formation is markedly increased and cellular injury occurs (Figure 1). Although mammalian cells express endogenous free radical scavenging enzymes, such as superoxide dismutase (SOD), catalase (CAT), and glutathione peroxidase (GPx), these antioxidative defenses are insufficient during MIR [3, 4]. Oxidative stress during MIR injury contributes to a vicious cycle as it promotes mitochondrial dysfunction, excitotoxicity, lipid peroxidation, and inflammation [5–7]. 

### 2.2. Sterile Inflammation

Ischemia and reperfusion cause sterile inflammation. Nevertheless, the consequences of MIR share many phenotypic parallels with activation of a host immune response directed toward invading microorganisms [8]. This sterile inflammation is mainly triggered by the interactions between toll-like receptors (TLRs) and their endogenous ligands generated in ischemic and reperfused myocardium, such as apoptotic cell debris, fibrinogen, high mobility group box (HMGB) 1, and heat shock proteins (HSPs) [9]. The activation of immune cell and cardiomyocyte TLR and other signaling pathways results in a vicious cycle of inflammatory response in the I/R region and causes significant cardiomyocyte apoptosis (Figure 1). Following the acute I/R period, the cardiac function is further compromised by adverse myocardial remodeling [10]. The magnitude of the inflammation during the acute phase determines the extent to which cardiac function is compromised during the following myocardial remodeling phase. 

During the sterile inflammation phase of MIR, TLRs play detrimental roles as demonstrated by extensive experimental evidence [11]. To date, 11 TLRs (TLR1–TLR11) have been identified in mammals. It should be noted that, during MIR, the expression of TLR4 is significantly increased in both the failing myocardium, and infiltrated macrophages and thus TLR4 is thought to be a central mediator of inflammation and cardiac injury. TLR4 has been identified as a mediator of inflammation and organ injury in several models of sterile tissue injury including MIR, and a soluble inhibitor of TLR4 was able to prevent contractile dysfunction in wild-type cells [12]. Using a temporary left anterior descending (LAD) artery occlusion model, Oyama et al. first observed myocardial infarct size reductions in 2 distinct strains of mice that lack functional TLR4 signaling, accompanied with reduced neutrophil infiltration in the affected myocardium [13]. TLR2, which is expressed in cardiomyocytes and many other cell types, also contributes to the pathogenesis of cardiac dysfunction during MIR [14, 15]. Activation of TLR2, TLR4, and TLR5 increases the myocardial level of the inflammatory cytokines, chemokines, and cell surface adhesion molecules [16]. Given the known role of TLR4 and TLR2 in MIR, inhibition of TLR4 and TLR2 signaling is a promising approach to reduce morbidity and mortality in MI patients.

There are a variety of TLR ligands generated during MIR. For example, heat shock proteins (HSPs) are a class of molecular chaperones that promote intracellular protein folding. They may be released into the extracellular space after cell trauma and interact with adjacent cells or distant cells via bloodstream delivery [17]. Extracellular HSP60 induced apoptotosis via the activation of TLRs [18]. Another example is HMGB1 which is a damage-associated molecular pattern (DAMP) protein secreted by injured cells [19]. It plays a major role in early MIR by binding to TLRs and the receptor for advanced glycation end products (RAGE), resulting in the activation of proinflammatory pathways and enhanced myocardial injury [20]. In fact, a prerequisite for neutrophil-mediated tissue damage is the “priming” effect of various pro-inflammatory stimuli generated by damaged tissue during MIR, such as HSP60 and HMGB1 [21]. Cytokines released by TLR-activated cells such as tumor necrosis factor-alpha (TNF-*α*) and IL-1 can elicit neutrophil polarization and upregulation of cell-surface glycoproteins such as macrophage adhesion molecule-1 (Mac-1) [22]; Mac-1 upregulation in peripheral neutrophils is a very early event in MIR [23].

### 2.3. Apoptosis and Mitochondrial Function

MIR leads to the activation of cell death programs, including apoptosis, autophagy-associated cell death, and necrosis [24]. Apoptosis involves an orchestrated caspase signaling cascade, including caspase-3 and caspase-9, which induces a self-contained program of cell death, characterized by the shrinkage of the cell and its nucleus, with plasma membrane integrity persisting until late in the process [25]. The balance between apoptotic factors Bcl-2 and Bax has been found altered in cardiomyocytes during ischemia [26]. Autophagy is stimulated by nutrient starvation and growth factor deprivation when cells are unable to take up external nutrients. Autophagy is also activated by decreases in ATP in order for the cell to maintain energy homeostasis and survival. Autophagy may serve primarily to maintain energy production during acute ischemia but switch to clear up damaged organelles during chronic ischemia or reperfusion [27].

Multiple cell signaling pathways, such as the AMPK, JNK, and NF-*κ*B pathways, have been shown to be involved in MIR-induced cardiomyocyte apoptosis (Figure 1). AMPK orchestrates the regulation of energy-generating and energy-consuming pathways; its activation has been shown to protect the heart against ischemic injury [28, 29]. Activated JNK signaling, especially in mitochondria, is associated with oxidative stress, mitochondrial dysfunction, and cell death [30]; it is a key modulation event in cell death mediated by reactive oxygen and nitrogen species [31]. JNK is also required for TNF-*α*-stimulated ROS production and cytochrome c-mediated cell death; Bcl-2 family members are essential components of this mitochondrial apoptotic machinery. Studies have suggested that blockage of JNK mitochondrial translocation or JNK inhibition prevents ROS production and mitochondrial dysfunction and may be an effective treatment for I/R-induced cardiomyocyte death [32–35]. The nuclear factor kappa B (NF-*κ*B) also modulates apoptosis during ischemia and reperfusion [36]. TLR signaling pathway leads to translocation of NF-*κ*B to the nucleus and thus up-regulation of expression of proinflammatory cytokines. However, there is the possibility that a crosstalk between the TLR/NF-*κ*B and PI3K/Akt signaling pathways and modulation of the crosstalk could protect the myocardium from I/R injury [37].

Within the mitochondria dependent intrinsic apoptosis pathway, which has an important function in cardiac cell injury under various pathological conditions [38], mitochondrial permeability transition pore (MPTP) opening plays a pivotal role [39]. The event of MPTP opening is affected by various factors including intracellular Ca^2+^, oxidative radicals, ATP levels and the levels of Bcl-2 family proteins [40].

### 2.4. Bone Marrow Stem Cell Migration

Bone marrow mesenchymal stem cells (BMSCs) are multipotent cells that secrete angiogenic factors. Injured tissues express specific receptors, such as CXCR4, and/or their ligands including stromal cell-derived factor-1 (SDF-1), to facilitate trafficking, adhesion, and infiltration of BMSCs. During MIR, BMSCs are preferentially attracted to and retained in the ischemic tissue [41, 42]. As a result of the hypoxic microenvironment, these BMSCs produce high levels of vascular endothelial growth factor (VEGF), leading to an increase in vessel density and facilitating myocardial regeneration and remodeling [43, 44] (Figure 1).

### 2.5. Angiogenesis

Angiogenesis refers to the sprouting, bridging, intussusception, and/or enlargement of capillaries. In the late stage of MI repair, enhancement of blood flow to ischemic myocardium can result from either true angiogenesis or the recruitment of preexisting coronary collaterals [45]. VEGF is an endothelial cell-specific angiogenic factor and also a critical regulator of angiogenesis that stimulates proliferation, migration, and proteolytic activity of endothelial cells [46]. Ischemia or coronary artery occlusion induces myocardial VEGF expression, which leads to an angiogenesis-induced restoration of tissue blood flow and the prevention of further tissue damage (Figure 1). In addition, VEGF is a potent survival factor during physiological and tumor angiogenesis, and has been shown to induce expression of anti-apoptotic proteins in endothelial cells [47, 48].

### 2.6. Other Factors

The activation of ATP-sensitive potassium (KATP) channel subunits and ATPase, and calcium (Ca^2+^) overload are also involved in MIR (Figure 1). Ischemia-reperfusion may activate some ion channels that do not open under normal physiological conditions. One such channel is the KATP channel, whose activation facilitates potassium ion efflux, hyperpolarization, and action potential repolarization. The resulting shortening of the action potential duration decreases the total influx of sodium and calcium, which alleviates overloading of intracellular calcium (Ca^2+^) which in turn weakens myocardial contraction force and reduces myocardial oxygen consumption. Therefore, the opening of KATP channels plays an active role in protecting the heart against MIR injury.

## 3. Effects and Mechanisms of Chinese Herbal Medicine in MIR

The typical symptoms of cardiovascular diseases induced by MIR have been recorded in several ancient books of Traditional Chinese Medicine (TCM), such as *Inner Canon of Huangdi *and *Treatise on Febrile Diseases*. In TCM, *Qi* (energy) and *Blood* (material) are the main components compromised in MIR, whereby the principal mechanism is considered to be a disorder or deficiency of *Qi* and a disorder of the circulation (blood stasis) that results in severe pain and even death. Therefore, the main aims of Chinese herbs and herbal formulations in MIR treatment are to regulate or replenish *Qi*, and to unblock circulation or resolve blood stasis. In Tables 1–4, we list four categories of Chinese herbal medicine that have been used in the practice of TCM and/or recent research, including compounds extracted from herbs (Table 1), single herbs (Table 2), decoctions (Table 3), and patent drugs made up of Chinese herbs (Table 4). All of the abbreviations used in these tables are listed at the end of the paper, and the main mechanisms and the representatives of Chinese herbal medicine in MIR treatment are schematized in Figure 1. In the following sections, these herbal medicines are grouped according to their efficacy in TCM terminology, and the underlying cellular and molecular mechanisms demonstrated by experimental investigations are discussed.

### 3.1. Anti-Oxidation

Many Chinese herbal medicines, including extracted compounds, single herbs, decoctions, and patent drugs, exert their beneficial effects on MIR via their anti-oxidative activity. A number of biomarkers have been used to evaluate the antioxidative effects of these Chinese herbal medicines, such as ROS, SOD, GPx, CAT, nitric oxide synthase (NOS), malondialdehyde (MDA), myeloperoxidase (MPO), heme oxygenase (HO)-1, superoxide anion, GOT, 15-F2t-isoprostane (15-F2t-IsoP), ET-1, cycloxygenase-2 (COX-2), thioredoxin-1 (Trx-1), thioredoxin-related protein-32 (TRP32), redox-sensitive PKC*ε*/mKATP pathway, glutathione (GSH), oxidized glutathione (GSSG), glutathione reductase (GRD), CuZn-superoxide dismutase (CuZn-SOD), and Mn-SOD. 

Through in vivo and in vitro experiments, Kim et al. revealed that palmatine, a compound extracted from the Chinese herb, *Coptidis rhizome*, markedly reduced serum MDA level, and the activity of SOD and CAT in the cardiac tissues, as well as the COX-2 and iNOS expressions in MIR myocardium of rats [49]. Jiang et al. reported that the MDA content and MPO activity in ischemic myocardial tissue of rats treated with Forsythoside B, a compound derived from the Chinese herb, *Lamiophlomis rotate *(Benth.)* Kudo*, were both significantly reduced. These reductions were accompanied by a significantly improved recovery in myocardial function [50]. Hwa et al. reported that 2-Methoxycinnamaldehyde (2-MCA), a compound derived from the Chinese herb, *Cinnamomum cassia,* significantly increased HO-1 induction by promoting the translocation of Nrf-2 from cytosol to nucleus in endothelial cells in an MIR model [51]. In addition, Hu et al. demonstrated that cyclovirobuxine D, a compound derived from the Chinese herb, *Buxus microphylla,* significantly protected rat aorta endothelial cells against hypoxia-induced injury and enhanced nitric oxide (NO) release from endothelial cells; these effects were inhibited by nitric oxide synthase (NOS) inhibitor N-nitro-L'argininemethyl ester (L-NAME) [52]. Das et al. studied the effects of a single herb, *Makhana*, and demonstrated that the cardioprotective properties of *Makhana* were linked to its ability to scavenge ROS [53]. Some decoctions and patent drugs made up of Chinese herbs have also been shown to exert the anti-oxidative effects on MIR. Zhao et al. found that *SiNi Decoction (SND),* composed of Chinese herbs, *Aconite, Ginger and Licorice,* could enhance the activity of myocardial and myocyte mitochondrial SOD and reduce MDA by increasing the expression of Mn-SOD mRNA [54]. Wang et al. reported that in rats treated with *Acanthopanax Senticosus Injection (ASI)* at doses of 25, 50, and 100 mg/kg via femoral vein infusion 30 min after coronary occlusion, the content of myocardial MDA was decreased significantly and dose-dependently and the activities of myocardial SOD and GSH-Px were increased dramatically [55].

### 3.2. Anti-Inflammation

The manifestation of MIR shares many phenotypic similarities with the activation of a host immune response directed toward invading microorganisms. HSPs and HMGB1 are both involved in the initiation of host defense and tissue repair. Molecules derived from immune cells and cardiomyocytes have been utilized as biomarkers to evaluate the anti-inflammatory effects of Chinese herbal medicine on MIR, including IL-6, MCP-1, TGF-*β*1, TNF-*α*, CRP, IL-1*β*, VCAM-1, ICAM-1, HMGB1, HSP25 and Hsp70, macrophage adhesion molecule-1 (Mac-1), troponinT (Tn-T), phosphorylated p38, activated MAPK, and tissue inhibitor of matrix metalloproteinase (TIMP)-1.

Ren et al. indicated that Tanshinone IIA (Tan IIA), a compound extracted from the Chinese herb, *Salvia miltiorrhiza Bunge*, attenuated expression of MCP-1, TGF-*β*1, and TNF-*α* as well as macrophage infiltration in rats when administered intragastrically at a dose of 60 mg/kg/day [56]. Jiang et al. reported that treatment with Forsythoside B significantly decreased the levels of TNF-*β*, IL-6, and HMGB1 in a rat MIR model [24, 50]. Results of a study by Chiu and Ko indicated that the reduction of Hsp25 and Hsp70 expression by Schisandrin B (Sch B), a compound extracted from Chinese herb, *Schisandra chinensis*, in MIR rats resulted in cardioprotection [57]. Shen et al. reported that neutrophils from MIR animals displayed a significant morphological change and Mac-1 up-regulation, both of which could be prevented by Tetrandrine (TTD), a compound extracted from the Chinese herb, *Stephania tetrandra *[23].

Decoctions and patent drugs made up of Chinese herbs have also been demonstrated to exert anti-inflammatory effects in MIR. Yin et al. showed that a significant reduction in TIMP-1 and TNF levels and improved cardiac function in MIR rats were achieved by treatment with *ShuMai Decoction* consisting of *Astragalus mongholicus* Bunge,* Salvia miltiorrhiza* Bge, and *Eupolyphaga sinensis, *in a dose-dependent manner [58]. Zhang et al. studied the patent drug *Xiongshao Capsule (XSC),* comprised of Chinese herbs, *Rhizoma Chuanxiong* and *Radix Paeoniae Rubra, *and found that it reduced levels of MCP-1 and TNF-*α* as well as inflammatory cell infiltration (ICI) in the ischemic myocardium [59].

### 3.3. Anti-Apoptosis

Alterations of pro and antiapoptotic signaling pathways, including changes in the levels of apoptosis-modulating molecules and induction of caspases, have been used to examine the anti-apoptotic effects of Chinese herbal medicine in MIR. Levels and/or activities of caspase-3, caspase-9, Bcl-2/Bax, p-JNK, p-AMPK, p-p38, phosphatidylinositol 3-kinase (PI3 K), Akt, p-I*κ*B-*α*, NF-*κ*B, p65, Bcl-2-associated X protein, cytochrome c, and forkhead transcription factor 3 (FOXO3) are among the commonly used biomarkers.

Sun et al. revealed that Salidroside and Tyrosol, two compounds extracted from the Chinese herb, *Rhodiola*, separately or in combination, significantly reduced caspase-3 activity, cytochrome c release, and JNK activation in an in vitro study [60]. Liu et al. reported that 3,5-Dimethoxy-4-(3-(2-carbonyl-ethyldisulfanyl)-propionyl)-benzoic acid 4-guanidino-butyl ester, derived from the Chinese herb, *Leonurus*, inhibited apoptosis by increasing the ratio of Bcl-2/Bax, decreasing the level of cleaved-caspase-3, and enhancing the phosphorylation of Akt [61]. An in vivo study by Jiang et al. demonstrated that rats treated with Forsythoside B showed a significant recovery in myocardial function due to down-regulated phosphorylation of IkB-*α* and NF-*κ*B [50]. 

Ling et al. studied the effects of the patent drug, *Cardiotonic Pill* (CP) combined with the Chinese herb, *Salvia miltiorrhiza, *and found that CP treatment (50 mg/mL) significantly inhibited TNF-*α*-induced apoptosis in cardiomyocytes through activating Akt signaling [62]. Others have showed that *Guan xin er hao (Guanxin II), *which consists of the Chinese herbs, *Safflower, red peony, salvia, Chuanxiong, and Dalbergiae Odoriferae*, tilted the balance between Bax and Bcl-2 toward an anti-apoptotic state, decreased mitochondrial cytochrome c release, reduced caspase-9 activation, and attenuated subsequent caspase-3 activation and postischemic myocardial apoptosis in rats [63, 64].

### 3.4. Protecting Mitochondrial Function

MPTP has been used as a target for protecting mitochondrial function by Chinese herbal medicine in the treatment of MIR. ATP-generation capacity, mitochondrial uncoupling, cAMP response element-binding protein (CREB), cytochrome c, cytochrome P-450, mitochondrial glutathione (GSH), mitochondrial Ca^2+^, and mitochondrial MDA have been used as biomarkers to evaluate the effects of Chinese herbal medicine.

Wong and Ko reported that a semipurified fraction of *Herba Cistanches* (HCF1) increased mitochondrial ATP-generation capacity and ADP-stimulated state respiration in H9c2 cardiomyocytes during MIR. HCF1 pretreatment could protect against MIR injury in rats presumably mediated by the induction of glutathione antioxidant [65]. Siu and Ko studied the single Chinese herb, *Cistanche,* and found it enhanced mitochondrial glutathione status, decreased mitochondrial Ca^2+^ level, and increased the mitochondrial membrane potential and respiration rate in rat hearts [66]. Others reported that the patent drug, *Guanxin II, *decreased mitochondrial cytochrome c release and attenuated caspase-3 activation in rat MIR myocardium [63, 64].

### 3.5. Increasing BMSCs Migration

Bone marrow mesenchymal stem cells (BMSCs) are preferentially attracted to and retained in ischemic tissue. SDF-1 and CXCR4 have been used as targets for increasing BMSC migration to protect cardiomyocytes against MIR. 

Tong et al. studied the effect of Tan IIA on MIR both in vitro and in vivo. Their data showed that combination treatment with Tan IIA and BMSCs significantly reduced the infarct size and improved cardiac function after MI, which primarily resulted from Tan IIA induced increase of the migration of BMSCs to ischemic region [67].

### 3.6. Promoting Angiogenesis

Angiogenesis limits MIR damage by restoring tissue blood flow. Related molecules such as VEGF, von Willebrand factor (vWF), hypoxia-inducible factor 1*α* (HIF-1*α*), VEGFR (Flt-1, KDR, and angiopoietin receptor (Tie-2)), platelet-derived growth factor (PDGF-BB), and phosphatidylinositol 3-kinase (PI3K) have been used as the targets for angiogenesis promotion to protect cardiomyocytes against MIR.

Xu et al. found that the compound, *Tanshinone IIA,* elicited a significant cardioprotective effect by up-regulating VEGF expression in MI rats and enhancing HIF-1*α* expression [68]. Experiments of Gao et al. showed that the expressions of vWF, HIF-1*α*, HIF-1*β*, and VEGF were significantly increased in myocardium treated with *Radix et Rhizoma Rhodiolae Kirilowii Decoction *[69].

### 3.7. Up-Regulating KATP Channel Subunits and ATPase, and Inhibiting Calcium Overload

KATP channel subunits Kir6.1, Kir6.2, SUR2A and SUR2B, Na^+^-K^+^-ATPase, Ca^2+^-ATPase and intracellular calcium (Ca^2+^), and L-type calcium current (I-CaL) have been used to assess the effects of Chinese herbal medicine in protecting cardiomyocytes against MIR. 

Han et al. examined the effects of Astragaloside IV (As IV), a compound extracted from the Chinese herb, *Astragalus membranaceus*. They found that As IV significantly up-regulated mRNA and protein levels of KATP channel subunits Kir6.1, Kir6.2, and SUR2A and SUR2B [70]. Lu and Zhao reported that Lycium barbarum polysaccharides, extracted from the Chinese herb, *Lycium barbarum, *significantly increased Na^+^-K^+^-ATPase and Ca^2+^-ATPase activities in myocardium of ischemia-reperfusion rats [71].

## 4. Summary and Perspective

In summary, significant progress has been made regarding the mechanistic research into the efficacy of Chinese herbal medicine for the treatment of MIR. However, much work remains. Most clinical studies were of limited extrapolatable value because of the small sample sizes and/or incomplete data. Experimental studies have focused mainly on single compounds extracted from Chinese herbs. Studies of Chinese decoctions or formulations are relatively scarce, although decoction and formulations are the main forms of therapy in TCM practice. Capitalization of the interactions between the different components and herbs is the essence of TCM. Many herbs are paired together to attenuate toxicity as well as to enhance efficacy. Encouragingly, the number of studies on patent Chinese herbs has been gradually increasing. These studies help us to understand the mechanisms underlying the use of Chinese herbs and formulations for the treatment of MIR. Accordingly, there is a strong likelihood that such ongoing research will lead to novel therapies for the treatment of myocardial ischemia and reperfusion injury using Chinese herbs and herbal formulations.

## Figures and Tables

**Table 1:** Efficacy and mechanisms of Chinese herb-derived compounds in the treatment of MIR.

Mechanism of action in TCM terminology	Plant	Compound	Mechanism	Biomarker/Targets	In vivo/In vitro	References
Tonifying *Qi* (energy) to activate circulation and enrich *Blood *	*Salvia miltiorrhiza *	Tanshinone IIA	Anti-inflammation	MCP-1, TGF-*β*1, TNF-*α*, NF-*κ*B	In vivo	[56]
			Antioxidant	VEGF, HIF-1*α*; MDA, SOD, GPx	Both	[68, 72]
			Antiapoptosis	Bcl-2/Bax, caspase-3	Both	[72]
			Promote angiogenesis	VEGF, HIF-1*α*	In vivo	[68]
			Promote BMSCs migration	SCF-1, CXCR-4	In vivo	[67]
		Sodium tanshinone IIA sulfonate	Anti-apoptosis	LDH, JNK, p38	In vivo	[73]
		Magnesium tanshinoate B	Anti-apoptosis	p-JNK, cytochrome c, caspase-3	In vitro	[74]
		Salvianolic acid A	Activate calcium channels	I-CaL	In vivo	[75]
		Salvianolic acid B	Promote angiogenesis	VEGF	In vivo	[76]
		Salvianolic acids	Antioxidant	15-F2t-IsoP, ET-1, CK-MB	In vivo	[77]
			Reduce ME	CK	In vitro	[78]
		Tanshinone combined with salvianolic acids	Inhibit of intracellular calcium, and anti-apoptosis, antioxidants	ICAM-1, Ca^2+^	In vivo	[79]
		Tanshinone IIA combined with salvianolic acid B	Antioxidant	CAT, L-arginine, eNOS, AMPK, Akt	In vivo	[80]
		Danshensu	Antioxidant, reduce ME	SOD, MDA; CKMB, LDH	In vivo	[81]
		Salvia miltiorrhiza extract	Antioxidant, reduce ME	MDA, SOD, and GPx; LDH, CK, GOT	In vivo	[82, 83]
		Aqueous extracts of *Salvia miltiorrhizae *	Reduce ME, promote angiogenesis	CK-MB and cTnT, 6-keto-PGF-1*α*/TXB-2	In vivo	[84]
		*Salvia miltiorrhizae *	Antioxidant	COX-2; TXB2, 6-keto-PGF1-*α*	In vivo	[85]
	*Radix Ginseng *	Saponin of red ginseng	Inhibit Ca^2+^ overload, up-regulate KATP	Ca^2+^; KATP; cTnI; PI3K	Both	[86, 87]
		Total ginsenosides	Antioxidant, anti-apoptosis	Ca^2+^; eNOS, iNOS, GR; PI3K, Akt	In vivo	[87, 88]
		Radix Ginseng extracts	Antioxidant, reduce ME	NO, eNOS; CK, LDH	In vivo	[88]
	*Astragalus membranaceus *	Astragaloside IV	Up-regulate KATP channel subunits, facilitate KATP currents	KATP channel subunits Kir6.1, Kir6.2, SUR2A, SUR2B	In vivo	[70]
	*Rhodiola *	Salidroside, tyrosol	Anti-apoptosis	caspase-3, p-JNK, cytochrome c	In vitro	[60]
	*Millettia pulchra *	17-Methoxyl-7-hydroxy-benzene-furanchalcone	Antioxidant, anti-inflammation, and anti-apoptosis	MDA; TNF-*α*; NF-*κ*B p65, Bcl-2-associated X protein	Both	[89]
	*Schisandra chinensis *	Schisandrin B	Anti-inflammation and	Hsp25, Hsp70	In vivo	[57]
			Antioxidant	cytochrome P-450	In vivo	[90]
	*Lycium barbarum *	*Lycium barbarum* polysaccharides	Increase Na^+^-K^+^-ATPase and Ca^2+^-ATPase, anti-apoptosis	Na^+^-K^+^-ATPase, Ca^2+^-ATPase; Bax, Bcl-2	In vivo	[71]

Moving *Qi* and activating circulation to resolve stasis	*Ligusticum wallichii *	Tetramethylpyrazine	Antioxidant, inhibit neutrophil	HO-1; Migrated neutrophil	In vivo	[91]
		Aqueous extracts of Rhizoma Chuanxiong	Reduce ME, promote angiogenesis	CK-MB, cTnT; 6-keto-PGF-1*α*/TXB-2	In vivo	[84]
	*Carthamus tinctorius* L.	Extracts of *Carthamus tinctorius *	Antioxidant, anti-inflammation	ROS, MDA, SOD; CRP, TNF-*α*, IL-1*β*; PI3K	Both	[92, 93]
	*Panax notoginseng *	Extracts of *Panax notoginseng *	Antioxidant, anti-inflammation	MDA, SOD; CRP, TNF-*α*, IL-1*β*	In vivo	[92]
		Notoginsengnosides	Reduce ME	CK	In vitro	[78]
	*Dipsacus asper *	Asperosaponin VI	Antioxidant, reduce ME, and protect mitochondrial function	SOD, GOT, GPx, MDA; CK-MB, LDH, cTnT; ICDH, MDH, *α*-KGDH, ATP, Ca^2+^	In vivo	[94]
			Anti-apoptosis, reduce ME	Bcl2/Bax, caspase-3; LDH, CREB, PI3K	In vitro	[95]
	*Pyrolae *	Flavonoid of Herba Pyrolae	Antioxidant, reduce ME	SOD, MDA; CK, LDH	In vivo	[96]
	*Lamiophlomis rotata *	Forsythoside B	Antioxidant, anti-inflammation	MDA, MPO, SOD, GPx;Tn-T, TNF-*α*, IL-6, HMGB1; IkBa, NF-*κ*B	In vivo	[50]
	*Sida cordifolia* L.	Hydroalcoholic extract of *Sida cordifolia* L.	Antioxidant, reduce ME	SOD, CAT; LDH, CK-MB	In vivo	[24]
	*Desmodium *	Desmodium gangeticum	Stimulate muscarinic receptors	Muscarinic receptor	In vivo	[97]

Inducing *Diuresis* to resolve stasis	*Leonurus *	3,5-Dimethoxy-4-(3-(2-carbonyl-ethyldisulfanyl)-propionyl)-benzoic acid 4-Guanidino-butyl ester	Anti-apoptosis	Caspase-3, Bcl-2/Bax, Akt	In vitro	[61]
		4-Guanidino-n-butyl syringate	Inhibit Ca^2+^ overload, antiapoptosis	Ca^2+^; Bcl-2, Bax, LDH	In vivo	[98]
	*Acorus gramineus *	*Acori graminei Rhizoma *	Inhibit calcium overload	Ca^2+^	In vivo	[99]
	*Phytolacca *	Oleanolic Acid	Anti-apoptosis	AMPK, p38, FOXO3	In vitro	[100]
	*Tetrandra *	Tetrandrine	Inhibit neutrophil, antioxidant	neutrophil adhesion, Mac-1; ROS	In vivo	[23]

Cooling *Blood* to stop bleeding	*Baicalensis *	Botanical Flavonoids	Antioxidant	ROS, NO, SOD, CAT, GPx	Both	[101, 102]
	*Coptidis rhizoma *	Palmatine	Antioxidant, reduce ME	SOD, MDA, COX-2; LDH, CK	In vivo	[49]
	*Buxus microphylla *	Cyclovirobuxine D	Antioxidant, reduce ME	KATP channel opening; NO, ROS, SOD, MDA; CPK, LDH, FFA	In vivo	[52, 103]

Tonifying *Qi* to invigorate *Yang *	*Cinnamon *	2-Methoxycinnamaldehyde	Antioxidant, anti-inflammation	VCAM-1,TNF-*α*, HO-1	In vivo	[51]
	*Herba Cistanches *	A semipurified fraction of Herba Cistanches	Protect mitochondrial function, antioxidant, and anti-apoptosis	ATP-generation, mitochondrial uncoupling; GSH; caspase-3	Both	[65]

Regulating *Qi* and moving *Qi *	*Corydalis *	Corydalis yanhusuo extract	Anti-apoptosis	Bax, Bcl-2	In vivo	[104]
	*Magnolia officinalis *	Magnolol	Antioxidant; inhibit neutrophil	MPO, superoxide anion; migrated neutrophil	In vivo	[105]

**Table 2:** Efficacy and mechanism of single Chinese herbs in the treatment of MIR.

Mechanism of action in TCM terminology	Herb	Mechanism	Biomarker/Targets	In vivo/In vitro	References
Replenishing and moving *Qi *	*Rhodiola *	Promote angiogenesis	VEGFR (Flt-1, KDR, and Tie-2)	In vivo	[106]
	*Euryale ferox (Makhana) *	Antioxidant	TRP32, ROS, and Trx-1	Both	[53]
	*Aurantii Fructus *	Recovery of contractile dysfunction	Perfusion pressure, aortic flow, and coronary flow	In vivo	[107]

**Table 3:** Efficacy and mechanisms of Chinese herb decoctions in the treatment of MIR.

Mechanism of action in TCM terminology	Decoction	Constituent herbs	Mechanism	Biomarker/Targets	In vivo/In vitro	References
Tonifying *Qi* to enrich *Blood *	*DangGui BuXue Decoction *	Astragali and Angelica roots	Antioxidant, protect mitochondrial function	GSH, GSSG, GRD	In vivo	[108]
	*BuYang HuanWu Decoction *	Astragalus, angelica, red peony, earthworm, and so forth	Reduce ME	LDH, CK, AST; CD40-CD40L	In vivo	[109]

Replenishing *Qi* to activate *Blood *and recover circulation	*ShuMai Decoction *	Astragalus mongholicus, Salvia miltiorrhiza, Eupolyphaga, Wallich and Hirudo nipponica Whitman, Moschus berezovskii	Anti-inflammation	TNF-*α*, p38, MAPK, TIMP-1	In vivo	[58]
			Promote angiogenesis	VEGF, PDGF-BB, PI3K, Akt	In vivo	[110]
Invigorating *Yang* to recover circulation	*Sini Decoction *	Aconite, ginger, and licorice	Antimitochondrial oxidation	SOD, MDA, MnSOD mRNA	In vivo	[54]

Moving *Qi* to activate circulation	*Dan-Chuan-Hong Decoction *	Salvia, Rhizoma Chuanxiong, and safflower	Anti-apoptosis	TUNEL	In vivo	[111]
Enriching *Blood *to engender fluid	*DanShen GeGen Decoction *	Radix Salvia miltiorrhiza, and Radix Puerariae lobatae	Antioxidant	Redox-sensitive PKC*ε*/mKATP pathway	In vivo	[112]

Enrich *Qi* and cool *Blood *	*Radix et Rhizoma Rhodiolae Kirilowii *	Radix, Rhizoma Rhodiolae kirilowii	Promote angiogenesis	vWF, VEGF, HIF-1*α*, HIF-1*β*	In vivo	[69]

**Table 4:** Efficacy and mechanism of patent drugs made up of Chinese herbs in the treatment of MIR.

Mechanism of action in TCM terminology	Patent drug name	Main ingredient	Mechanism	Biomarker/Targets	In vivo/In vitro	References
Activating *Blood* to resolve stasis, and moving *Qi* to relieve pain	*XinKeShu Tablet *	Salvia, arrowroot, woody, hawthorn, panax	Antioxidant, promote angiogenesis	eNOS; VCAM-1	In vivo	[113]
	*TongXinLuo Superfine *	Ginseng, leeches, scorpion, Eupolyphaga, centipede, et al	Antioxidant, proangiogenesis	NO, eNOS; vWF, Hhcy	In vivo	[114]
	*ShuMai Capsule *	Peanut shells	Promote angiogenesis	vWF, VEGF	In vivo	[115]

Moving *Qi* to activate circulation and relieve pain	*GuanXin ErHao (Guanxin II) *	Safflower, red peony, salvia, Chuanxiong, and so forth	Anti-apoptosis, protect mitochondrial function	Caspase-3, Caspase-9, Bcl-2/Bax, cytochrome c, Akt	In vivo	[63, 64]
	*XiongShao Capsule *	Rhizoma Chuanxiong, Radix Paeoniae Rubra	Anti-inflammation	TNF-*α*, MCP-1, ICI	In vivo	[59]

Replenishing *Qi* to activate circulation, and moving *Qi* to relieve pain	*Vigconic 28 (VI-28) *	Radix Ginseng, Cornu Cervi, Cordyceps, Radix Salviae, Semen Allii, and so forth	Antioxidant, protect mitochondrial function	GSH, *α*-TOC, CuZn-SOD, Ca^2+^-induced permeability, mitochondrial MDA, Ca^2+^, cytochrome c	In vivo	[116]
	*Acanthopanax Senticosus Injection *	Acanthopanax	Antioxidant, inhibit Ca^2+^ overload	SOD, MDA, GPx; Ca^2+^	In vivo	[55]
	*ShuangShen NingXin Capsule *	Ginseng total saponins, total salvianolic, corydalis	Antioxdiant	SOD, MDA	In vivo	[117]
	*ShuangShen TongGuan Recipe *	Ginseng, astragalus, Atractylodes, and so forth	Anti-inflammation	NF-*κ*B, p65, TNF-*α*, ICAM-1, MJIC-Cx43	In vivo	[118]

Replenishing *Qi* and invigorating *Yang *	*ShenFu Injections *	Red ginseng, Monkshood	Antioxdiant, reduce ME, up-regulate-ATPase	SOD, GPx; LDH, CK; Na^+^-K^+^-ATP and Ca^2+^-ATP	In vivo	[119]
						
Replenishing *Blood *and activating circulation	*DanHong Injection *	Salvia, safflower	Antioxidant	SOD, MDA	In vivo	[120]
	*Cardiotonic Pill *	Salvia miltiorrhiza	Anti-apoptosis	Caspase-3, Akt	In vivo	[62]

## Abbreviations

ROS: Reactive oxygen species
NOS: Nitric oxide synthase
MDA: Malondialdehyde
SOD: Superoxide dismutase
GSH: Glutathione
GPx: Glutathine peroxidease
MPO: Myeloperoxidase
CAT: Catalase
COX-2: Cycloxygenase-2
GOT: Glutamic oxalacetic transaminase
ME: Myocardial enzymes
TRP32: Thioredoxin-related protein-32
GSH: Glutathione
GSSG: Oxidized glutathione
GRD: Glutathione reductase
CuZn-SOD: CuZn-superoxide dismutase
PI3K: Phosphatidylinositol 3-kinase
HMGB1: High-mobility group box1
HSP: Heat shock protein
TIMP: Tissue inhibitor of matrix metalloproteinase
ICI: Inflammatory cell infiltration
LDH: Lactate dehydrogenase
CK: Creatine kinase
CK-MB: Creatine kinase isoenzyme-MB
TXB2: Thromboxane B2
VEGF: Vascular endothelial growth factor
HIF-1a: Hypoxia-inducible factor 1a
Vwf: Von Willebrand factor
SDF-1: Stromal cell-derived factor-1
SCF-1: Stem cell factor-1
CXCR4: CXC chemokine receptor 4
I-CaL: L-type calcium current
15-F2t-IsoP: 15-F2t-isoprostane
6-keto-PGF1-a: 6-keto-prostaglandin F1alpha
GR: Glucocorticoid receptor
CREB: cAMP response element-binding protein
Tn-T: TroponinT
FOXO3: Forkhead transcription factor 3
Mac-1: Macrophage adhesion molecule-1
HO-1: Heme oxygenase-1
Tie-2: Angiopoietin receptor
TRP32: Thioredoxin-related protein-32
Trx-1: Thioredoxin-1
GSSG: Oxidized glutathione
GRD: Glutathione reductase
PDGF-BB: Platelet-derived growth factor
Hhcy: Hyperhomocysteinemia
MJIC-Cx43: Myocardial junction intercellular communication connexin 43.
